# Pharmacological targeting of smoothened receptor cysteine-rich domain by Budesonide promotes *in vitro* myelination

**DOI:** 10.3389/fnmol.2024.1473960

**Published:** 2024-10-15

**Authors:** Antonella Damiana Recchia, Alessandra Dominicis, Vincenzo Maria D'Amore, Tommaso Fabiano, Aland Ibrahim Ahmed Al Jaf, Simone Peria, Francesco Basoli, Alberto Rainer, Luciana Marinelli, Francesco Saverio Di Leva, Antonella Ragnini-Wilson

**Affiliations:** ^1^Department of Biology, University of Rome “Tor Vergata”, Rome, Italy; ^2^Department of Pharmacy, University of Naples “Federico II”, Naples, Italy; ^3^Department of Life Sciences, University of Trieste, Trieste, Italy; ^4^Department of Science and Technology for Sustainable Development and One Health, Università Campus Bio-Medico di Roma, Rome, Italy; ^5^Department of Engineering, Università Campus Bio-Medico di Roma, Rome, Italy; ^6^Fondazione Policlinico Universitario Campus Bio-Medico di Roma, Rome, Italy

**Keywords:** remyelination, oligodendrocytes, glucocorticoids, molecular docking & molecular dynamics (MD) simulation, glia cells, hedgehog (Hh), OPC, drug mechanism of action

## Abstract

**Background:**

The myelin sheath ensures efficient nerve impulse transmission along the axons. Remyelination is a spontaneous process that restores axonal insulation, promoting neuroprotection and recovery after myelin damage. There is an urgent need for new pharmacological approaches to remyelination and to improve the most effective molecules. Some glucocorticoids (GC) were identified through phenotypical screens for their promyelinating properties. These GC compounds share the ability to bind the Smoothened (Smo) receptor of the Hedgehog (Hh) pathway. Gaining a deeper insight into how they modulate Smo receptor activity could guide structure-based studies to leverage the GCs’ potent promyelinating activity for a more targeted approach to remyelination.

**Methods:**

Here we focused on clarifying the mechanism of action of Budesonide, a GC known to bind the Smo cysteine-rich domain (CRD) and prevent Smo translocation to the cilium in fibroblasts. Our study employed a combination of cellular, biochemical and molecular dynamics approaches.

**Results:**

We show that treating oligodendroglial cells with Budesonide promotes myelination of synthetic axons and reduces Smo CRD conformational flexibility. This inhibits the Smo-mediated canonical signaling while activating the Liver Kinase B1 (LKB1)/ AMP-activated protein kinase (AMPK) pathway, leading to Myelin basic protein (MBP) expression.

**Discussion:**

These insights pave the way for pharmacological targeting of Smo CRD to enhance oligodendrocyte precursor cells (OPCs) differentiation and improve remyelination.

## Introduction

Throughout adulthood, the central nervous system (CNS) myelin sheath can be damaged by environmental factors, injuries, infections, and the progression of demyelinating diseases. The damaged myelin causes inflammation and neurodegeneration, leading to severe consequences on affected patients’ motor and intellectual abilities. Remyelination of injured axons is a spontaneous process that declines with age and is reduced or absent in patients with multiple sclerosis (MS). A growing body of evidence shows that the process of remyelination can be pharmacologically stimulated (Deshmukh et al., 2013; Mei et al., 2014, 2016; Najm et al., 2015; Porcu et al., 2015; Del Giovane et al., 2022). Pathophysiological studies have shown that promyelinating compounds promote the proliferation of neural precursor cells (NPCs) and/or oligodendrocyte (OL) precursor cells (OPCs) and their differentiation in myelinating OLs (Balestri et al., 2021; Caprariello and Adams, 2022). Although some compounds have entered clinical studies, their effectiveness in humans has yet to be proven (Allanach et al., 2022; Bebo et al., 2022). The devastating consequences of CNS demyelination on patients’ health and quality of life, highlight the urgent need for a deeper understanding of how promyelinating drugs promote CNS remyelination (Bebo et al., 2022).

Network analyses were conducted on the targets of drugs that promote remyelination to identify potential hub proteins involved in regulating OPCs differentiation and remyelination (Nocita et al., 2019; Caprariello and Adams, 2022; Sax et al., 2022). These analyses revealed that the largest cluster of drugs promotes the accumulation of 8,9-unsaturated sterols by inhibiting three enzymes of the cholesterol biosynthetic pathway, namely Emopamil binding protein (EBP), Sterol 14-demethylase (CYP51) and Sterol C-14 reductase (S14R) (Hubler et al., 2018; Sax et al., 2022). A second group of drugs, including glucocorticoids (GCs) and drugs that bind directly to the Smoothened (Smo) receptor of the Hedgehog (Hh) pathway biosynthesis (Najm et al., 2015; Porcu et al., 2015; Vicario et al., 2019; Del Giovane et al., 2022), have no major effects on cholesterol biosynthesis (Sax et al., 2022). A third group is more heterogeneous and includes hormones, vitamin D, retinoic acid and agonists or inhibitors of OPCs differentiation (Balestri et al., 2021). This group of drugs might also share the ability to affect enzymes of cholesterol biosynthesis as a secondary target (Caprariello and Adams, 2022).

It is widely believed that gaining a deeper understanding of how OPCs transition from proliferating to differentiating could lead to advancements in research for developing remyelination drugs. Pathophysiological studies show that in the event of myelin damage, chemotactic factors released by surrounding cells and activated macroglia prompt OPCs in the affected area to start repairing the myelin. Neural stem cells (NSCs) located in niches in the subventricular zone (SVZ) respond to these signals by multiplying, moving to the damaged area, and differentiating into OPCs, ultimately helping to restore the myelin in a second wave (Codega et al., 2014; Yeung et al., 2014; Duncan et al., 2018). The differentiation of OPCs into OLs requires the carefully timed and sequential activation of specific sets of transcription factors, such as Myelin regulatory factor (MyRF) and SRY-box transcription factor 10 (SOX10; Emery et al., 2009). The inhibition of genes that hinder OPCs differentiation, including G protein-coupled receptor 17 (GPR17) and the Glioma-associated oncogene family zinc finger 1 (Gli 1) (Gregath and Lu, 2018; Radecki et al., 2020; Hughes and Stockton, 2021).

The role of Gli1 as an effector of the Smo receptor in the Sonic Hedgehog (Shh) pathway is well-established. However, there has been an ongoing debate about the significance of Shh/Smo canonical signaling in the maturation of OPCs to OLs (Radecki et al., 2020; Fang et al., 2022; Nocera et al., 2024). It is well described that Shh uprising in the SVZ stimulates NPCs proliferation (Samanta et al., 2015; Radecki and Samanta, 2022). At the molecular level, the binding of Shh to the protein Patched homolog 1 (Ptch1) relieves Smo from Ptch1 inhibition, allowing its activation and migration from endosomes to the cilium (Rana et al., 2013). Smo is a 7-pass transmembrane protein belonging to the Frizzled (class F) family of G protein-coupled receptors (GPCRs) that in the so-called “canonical” pathway stimulates the oncogene proteins Gli1-3. Since Smo-mediated activation of Gli1 positively regulates the transcription of genes involved in proliferation, this signal promotes NPCs proliferation. On the contrary Smo inhibition stimulates NPCs differentiation towards the OPCs lineage by preventing Gli1 expression (Samanta et al., 2015; Namchaiw et al., 2019; Radecki et al., 2020; Del Giovane et al., 2022; Nocera et al., 2024).

Following the idea that stimulating “non-canonical” signalling through drug binding to Smo could promote OPC differentiation, we identified the quinolone GSA-10 ([propyl 4-(1-hexyl-4-hydroxy-2-oxo-1,2-dihydroquinoline-3-carboxamido) benzoate]) as potent promyelinating agent (Del Giovane et al., 2022). This drug, originally developed to bind the Smo agonist (SAG) binding domain in Smo (Gorojankina et al., 2013; Manetti et al., 2016), has shown properties in promoting Oli-neuM oligodendroglia differentiation till synthetic axon wrapping. Our research proved that treating mice with GSA-10 after lysolecithin-induced demyelination stimulates OPC recruitment to the demyelinated area of the corpus callosum. OPC differentiation in myelinating OL is accompanied by Gli1 downregulation (Del Giovane et al., 2022). In line with these findings, treatment with GSA-10 or Clobetasol of the Oli-neuM oligodendroglia mouse cell line does not activate the Smo-mediated Gli1 signaling pathway (Nocita et al., 2019; Del Giovane et al., 2022). These findings are in the line of pieces of evidence showing the requirement of Gli1 downregulation for NPC-fating to the OPC lineage (Radecki et al., 2020; Radecki and Samanta, 2022; Samanta et al., 2015) and support the view that drugs binding to Smo and inhibiting Gli1 activity could be effective in promoting myelination.

Given that Smo can activate various signaling pathways resulting in different cellular effects such as osteogenesis, apoptosis and ciliogenesis (Akhshi et al., 2022) in addition to OPC differentiation, there is a broader question to be answered regarding how different ligands interact with Smo’s cysteine-rich domain (CRD) or transmembrane domain (TMD) and regulate its intracellular responses and functions (Fang et al., 2022; Hu et al., 2022).

To get insights into how the binding of GCs to Smo can stimulate OPCs differentiation and remyelination we focused on Budesonide, a GC that was previously shown to bind to the Smo CRD (Wang et al., 2012; Rana et al., 2013). We conducted parallel molecular dynamics simulations and functional studies to investigate how Budesonide affects the structure of Smo and its impact on the ability of Oli-neuM cells to differentiate until they engage synthetic axons. Additionally, we studied the impact of Budesonide treatment on Oli-neuM oligodendroglia mouse cells, comparing those with and without Smo receptor expression. Our focus was on its influence on Myelin Basic Protein (MBP) expression and in Smo-mediated signalings.

In this work, we have clarified in oligodendroglia cells that Budesonide by binding to Smo CRD inhibits Gli1 gene expression and results in the induction of the expression of MBP. We show that the signaling initiated by Budesonide involves the activation of Liver Kinase B1 (LKB1)/AMP-activated protein kinase (AMPK). Our detailed molecular dynamics simulations have focused on understanding the atomic-level effects of Budesonide binding to the Smo CRD. These simulations have been conducted in parallel with our functional studies and have revealed that Budesonide’s unique regulatory activity on Smo is due to the orientation of the CRD domain in relation to the TM, which in turn influences its allosteric communication with the TM5 and TM6 helices.

These findings enhance our knowledge in the development of promyelinating drugs by suggesting that Smo inhibition via drug binding to the CRD could be exploited to identify drugs that promote remyelination.

## Materials and methods

### Cell culture and media

The Oli-neuM line (Cellosaurus ExPASy CVCL_VL76) was grown in either growth medium (GM) or differentiation medium (DM) containing 500 μg/mL Geneticin (G418, Gibco^™^, Thermo Fisher Scientific, Waltham, MA, United States, 10131027) and maintained at 37°C in 5% CO_2_, as previously described (Porcu et al., 2015).

### Compounds treatment

Budesonide (Selleckchem.com, S1286, Cologne Germany) was dissolved in 100% Dimethyl sulfoxide (DMSO; A3672, AppliChem, Damstad, Germany) and used at the final concentrations indicated in the text. Clobetasol (Prestw-781) was purchased from Prestwick Chemical Library^®^, Illkirch-Graffenstaden, France and used at a final concentration of 10 μM as previously described (Porcu et al., 2015). Dorsomorphin (S7306, Selleckchem, 50,829 Cologne Germany), a selective inhibitor of AMPK (Meley et al., 2006), was purchased from Selleckchem.com, the stock concentration was 100 mM, and it was employed at a concentration of 3 μM, previously demonstrated as the working concentration in Oli-neuM cell line (Del Giovane et al., 2022). 0.5% DMSO max was added to the vehicle treatment in all experiments. Unless otherwise stated, drugs were administrated in DM for 48 h to cells left to grow in GM for 24 h. Culturing and time of drug treatments have been established previously based on the timing and concentration for optimal MBP expression in Oli-neuM (Meley et al., 2006; Porcu et al., 2015; Neumann et al., 2019; Del Giovane et al., 2022). Engagement tests were performed as previously described in growth chambers containing aligned polystyrene (PS) microfibers of 2–4 μm manufactured and used as previously described (Nocita et al., 2019). For microfiber engagement analysis, the cells were treated for at least 72 h, according to previously established protocols (Del Giovane et al., 2022; Dominicis et al., 2023).

### Quantitative immunofluorescence microscopy analysis

Cells were seeded in 96-well plates (Grainer bio-one, Kremsmünster, Austria, 655,090 CHIMNEY WELL, μCLEAR^®^, NERO, CELLSTAR^®^, TC) pretreated with 10 μg/mL fibronectin (F0895; Sigma-Aldrich, Burlington, MA, United States) after 48 h with treatment in DM were fixed and processed for immunofluorescence (IF), as previously described (Nocita et al., 2019; Del Giovane et al., 2022). The automatized acquisition was performed using a Leica DMI6000 B epifluorescence inverted microscope (Leica Microsystems, Wetzlar, Germany), equipped with Leica Application Suite X and Matrix Screener software (version 3.0) at 20× magnification (HCX PL FLUOTAR 20 × NA 0.4). Images were then quantified and statistically analyzed with ScanR Analysis software (version 1.1.0.6 or 3.0; Olympus, Tokyo, Japan), as previously described (Porcu et al., 2015; Nocita et al., 2019). Hoechst 33342 (H3570, Thermo Fisher Scientific Inc., Waltham, MA, United States) and phalloidin (A12380; 1:40; Thermo Fisher Scientific Inc., Waltham, MA, United States) staining were performed to detect nuclei and actin cytoskeleton. Rat anti-MBP (MCA409S, 1:100; AbD Serotec, Hercules, CA, United States), was used as primary antibody (Ab) and Alexa Fluor 488 as secondary Ab (Thermo Fisher Scientific Inc., Waltham, MA, United States), as indicated in the text. Image analysis was performed as previously described (Porcu et al., 2015).

### Evaluation of cell engagement in PS microfibers

Cell culture chambers containing electro-spun PS microfibers were prepared as indicated in Nocita et al. (2019), UV-sterilized before use, and pre-treated with 10 μg/mL fibronectin (F0895; Sigma-Aldrich, Burlington, MA, United States). 60,000 Oli-neuM cells were seeded in GM. After 24 h, the medium was exchanged for either DM supplemented with 0.5% DMSO (vehicle) or the indicated drugs. After 72 h at 37°C in 5% CO_2_, cells were processed for fixation and IF microscopy analyses. Acquisition and engagement analyses were performed as described in Nocita et al. (2019). Confocal images were acquired as previously described (Nocita et al., 2019) with a 40X objective, and 3D volume reconstruction was performed using Imaris software (Bitplane AG, Zúrich-Switzerland). Quantification of % of engagement was performed by analyzing 75 images per sample for each treatment in 3 biological replicates (*n* = 3). The percentage of engaged cells was estimated by counting the total number of nuclei within a range of 86 μm from the fiber. Cells were considered engaged if nuclei were touching the fiber. The mean data (± SEM) of three biological replicates (3n) were plotted on the graph using Graph Pad (version 7).

Hundred engaged cells for each sample of each biological replicate were randomly selected to measure the mean values of the length of the membrane extensions (processes) along the PS fibers. The length of the processes extending along the fibres (μm) was measured by using the Analyses/Measure tool of ImageJ (v.1.54d). The variability (SEM) observed among biological replicates indicated that the measurement of 100 cells for each biological replicate is sufficiently representative of the group variability for this parameter.

### Total RNA extraction and qPCR

125,000 Oli-neuM cells/well were seeded in 12-well plates. After 48 h, GM was substituted with DM, to which treatment or vehicle was added. After 48 h, RNA extraction, quantification, and cDNA production were performed using RNA-Solv Reagent (R6830-01; VWR, Radnor, PA, United States). 2 μg of the RNA per sample was retro-transcribed following the High-Capacity cDNA Reverse Transcription Kit (4,368,814; Thermo Fisher Scientific Inc., Waltham, MA, United States) manufacturer’s instruction. Quantitative polymerase chain reaction (qPCR) was performed using SYBR Green Technology and the QuantStudio R 3 Real-Time PCR System (Applied Biosystems R, Thermo Fisher Scientific Inc., Waltham, MA, United States). Primer pairs used with StoS Quantitative Master Mix 2X SYBR Green-ROX (GeneSpin Srl, Milan, Italy) are indicated in Table 1. Specifically, *Gapdh* was used as an endogenous control to normalize data. 50 ng of cDNA per sample was used per reaction. qPCR was performed in triplicate in MicroAmp Fast Optical 96-Well Reaction Plate (Applied Biosystems R, Waltham, MA, USA). The 2^−ΔΔCT^ relative quantification method was used to determine fold change in expression. This consists of two main steps: normalization of threshold cycle (CT) values of the target mRNAs to the CT values of the endogenous control *Gapdh*, in the same samples (ΔCT = CT target–CT *Gapdh*) and further normalization to the control (ΔΔCT = ΔCT–ΔCT vehicle). The fold change in expression was then obtained as log_2_(2^−ΔΔCT^) and represented in the plots.

**Table 1:** Primer sequences used for qRT-PCR.

Gene name	Forward (5′-3′)	Reverse (5′-3′)
*Gapdh*	CCAATGTGTCCGTCGTGGATCT	GTTGAAGTCGCAGGAGACAACC
*Gli1*	CCCATAGGGTCTCGGGGTCTCAAA	GGAGGACCTGCGGCTGACTGTGTAA
*Mbp*	TACCCTGGCTAAAGCAGAGC	GAGGTGGTGTTCGAGGTGTC
*Plp*	GGCTAGGACATCCCGACAAGT	GGCAAACACCAGGAGCCATA
*Mal*	CAGATCCCATCATCAGCCCC	TGGCTGTGTTAAGTGGGCAA

### Crude extract preparation and immunoblot analysis

Typically, 2.75 × 10^5^ Oli-neuM cells were seeded in 6-well plates in GM media, until 70% confluence. Unless otherwise specified, cells were treated for 48 h in DM with the drugs indicated in the text. For immunoblot analyses the following Abs, diluted in TBS and 4% BSA, were used: anti-AMPKa1/2 (Santa Cruz Biotechnology, sc-74461), anti-p-AMPKa1/2 (Thr172) (Santa Cruz Biotechnology, sc-33524). Cell extract (CE) preparation and immunoblot analyses were performed as previously described (Porcu et al., 2015; Nocita et al., 2019). The band signal was detected by ChemiDoc™ Imaging System 12,003,153 (Bio-Rad) and intensity was estimated using the ImageJ 1.54d. The data were plotted on a graph using GraphPad Prism 7.0 (GrahPad Software, San Diego, CA, United States) as fold change versus vehicle, arbitrarily set to 1.

### Immunoprecipitation

The Oli-neuM cells were typically seeded on Petri dishes and allowed to grow in GM for 24 h prior to media replacement with DM. The treatments are indicated in the text and were applied in DM for 48 h. The crude extract was obtained by scraping the plate with a Lysis Buffer (25 mM Tris–HCl pH 7.4, 150 mM NaCl, 1 mM EDTA, 1% NP-40, 5% glycerol, 0.1 μL/mL PMSF and 10 μL/mL PIM). Cells were lysed and total protein extraction was quantified using spectrophotometer Thermo Scientific NanoDrop™ 1,000 Spectrophotometer (Thermo Fisher Scientific Inc.; USA). To remove the non-specific binding 1 mg of total lysate was then incubated in a wheel with the Protein G PLUS-Agarose (Santa Cruz Biotechnology, sc-2002) beads for 90′ at 4°C. After centrifugation at 500 x g for 5′, the supernatant was removed and incubated at 4°C overnight in a wheel with 2 μg of anti-LKB1 Ab (Santa Cruz Biotechnology, sc-32245). Samples were further incubated with the Protein G PLUS-Agarose (Santa Cruz Biotechnology, sc-2002) beads for 2 h at 4°C and after centrifugation the immunoprecipitation (IP) pellet was resuspended in Washing Buffer (50 mM Tris–HCl pH 7.4, 150 mM NaCl, 5 mM EDTA, 1% NP-40, 5 mM MgCl_2_, 0.1 μL/mL PMSF and 10 μL/mL PIM) three times before to SDS-loading buffer addition, meanwhile, the IP supernatant was kept at −20°C till SDS-PAGE analyses. IP pellets and IP supernatant were then mixed with SDS-loading buffer (6X) and incubated at 95°C for 5′. After SDS-PAGE electrophoresis western blotting and immunodetection were performed as previously described using Anti phosphorylated LKB1 Ab (Santa Cruz Biotechnology, sc-271924). Results were quantified on bands using ImageJ 1.54d.

### Statistical methods

In studies performed in multiwell plates (IF and qPCR), three experimental replicates are present in each plate, and the mean values obtained from data analysis were considered as one biological replicate. The effects of each drug treatment ratioed its internal control (vehicle) in IF experiments, WB, and qPCR data were analyzed to determine statistically significant differences among multiple single or combined treatments by paired two-tailed Student’s *t*-test and one-way analysis of variance (ANOVA) with Tukey’s tests, respectively. For IF quantitative analyses 25 images per well were acquired and the mean values of at least three wells (one biological replicate) were normalized toward an internal control, typically the vehicle (DM+ DMSO 0.5% max). The mean values ± SEM of three biological replicates were calculated and plotted on the graph using GraphPad Prism version 7.00 for Windows (GraphPad Software, Boston, Massachusetts USA, www.graphpad.com). Statistical analyses were performed using GraphPad Prism tools two tailed t test and one-way ANOVA multiple comparisons.

### Molecular docking

Docking calculations were performed using the 3D structure of human Smo (hSmo) in complex with cholesterol (PDB code: 5L7D, Byrne et al., 2016). This choice was driven by the chemical similarity between cholesterol and the investigated ligand, Budesonide. Notably, in this structure, Smo is in an inactive conformation, suitable for docking compounds with pharmacological activity like Budesonide, due to mutations which were reverted to the wild-type amino acids prior calculations. The receptor conformation was prepared using the Protein Preparation Wizard tool, implemented in the Maestro Suite 2021 (Madhavi Sastry et al., 2013). Any missing residues were added and conformationally optimized using the Prime toolkit (Jacobson et al., 2002, 2004). The missing intracellular loops (ICL) 2 and 3 were built for homology using the PDB structures 5L7I (Byrne et al., 2016) and 4JKV (Wang et al., 2013) as templates, respectively. Correct bond orders were assigned, missing hydrogen atoms were added, and all water molecules were deleted from the receptor structure. Protonation and tautomeric states at pH 7.4 were assigned to the side chains using Epik (Johnston et al., 2023). Finally, the positions of all the hydrogens were minimized. The docking search box (20 Å × 20 Å × 20 Å) was centered around the position of cholesterol in the experimental complex. Docking calculations were performed employing the Glide (version 9.3) SP protocol and the OPLS3A force field.

### Molecular dynamics

Prior to Molecular Dynamics (MD) simulation, the Smo N-terminus (P57) and C-terminus (W549) were capped with acetyl and N-methyl groups, respectively. For comparison, the apo-Smo system was obtained by removing the ligand from the same structure used for docking Budesonide (PDB code: 5L7D) and following the same preparation protocol described in the previous paragraph. For each system, the receptor was then embedded in a 110 Å x 110 Å (along the x and y axes) pre-equilibrated 1-palmitoyl-2-oleoylphosphatidylcholine (POPC)—cholesterol (7:3 molar ratio) bilayer and solvated using the TIP3P water model with the aid of the membrane-builder tool of CHARMM-GUI.org. The ff14SB (Maier et al., 2015) and lipid17 Amber force fields were used to parametrize the protein and the lipids, respectively. The bonded and van der Waals parameters for Budesonide were taken from the GAFF force field (Wang et al., 2004), whereas its atomic partial charges were computed using the two-stage restrained electrostatic potential (RESP) fitting procedure (Bayly et al., 1993) implemented in Antechamber (Wang et al., 2006). The required ESP was calculated using the Gaussian package (Frisch et al., 2009) at the Hartree-Fock level of theory with the 6-31G* basis set, following a geometry optimization performed with the B3LYP functional.

The GROMACS 2020.6 code (Berendsen et al., 1995) was used to perform the simulations. A cutoff of 12 Å was used for short-range interactions. The long-range electrostatic interactions were computed using the particle mesh Ewald method with a 1.0 Å grid spacing under periodic boundary conditions. The non-iterative LINCS (Hess et al., 1997) algorithm was applied to constrain bonds, allowing the use of a 2 fs integration time step. To resolve all steric clashes, each system underwent 30,000 steps of steepest descent energy minimization in three phases. In the first phase, the system’s heavy atoms were kept fixed to relax only the hydrogens and water molecules; during the second phase, the lipid bilayer was also released; and in the third phase, all atomic positions were minimized. Each complex was then equilibrated and heated to 300 K, alternating NPT and NVT cycles (for a total of 30 ns) with the Berendsen coupling bath and barostat (Berendsen et al., 1984), while gradually decreasing harmonic constraints on the heavy atoms of the membrane, protein, and ligands. During the production runs, the pressure of 1 atm and the temperature of 300 K were maintained with the stochastic velocity rescaling (Berendsen et al., 1984) and the Parrinello and Rahman (1981) algorithms, respectively. The stability of the MD simulated system was assessed by monitoring the protein RMSD with respect to the starting conformations over simulation time (Supplementary Figure S1).

### Protein structure network analysis

Network parameters such as hubs, communities, and structural communication analyses were obtained by using the WebPSN 2.0 web-server (Felline et al., 2020).

The methodology builds the Protein Structure Graph (PSG) based on the interaction strength of two connected nodes, according to Equation 1:


(1) $$I_{i,j}=\frac{n_{ij}}{\sqrt{N_{i}N_{j}}}100$$

where interaction percentage (***I***_***ij***_) of nodes ***i*** and ***j*** represents the number of pairs of side-chain atoms within a given cut-off value (4.5 Å), while ***N***_***i***_ and ***N***_***j***_ are normalization factors. The interaction strength (represented as a percentage) between residues ***i*** and ***j*** (***I***_***ij***_) is calculated for all node pairs. If ***I***_***ij***_ is more than the minimum interaction strength cutoff (***I***_***min***_) among the residue pairs, then it is considered to be interacting and hence represented as a connection in the PSG.

## Results

### Budesonide promotes oligodendrocyte differentiation

The O4 marker is found on the surface of oligodendrocyte progenitors and is commonly used as the earliest recognized marker specific for the oligodendroglial lineage. Previous research showed that Budesonide can promote O4 expression in the Oli-neu mouse oligodendroglia cell line (Joubert et al., 2010). However, further studies have not yet addressed whether Budesonide treatment can promote OPC differentiation till axon remyelination. This latter step is essential for drug efficacy in remyelination. To investigate the Budesonide potential for remyelination therapies, we used the Oli-neuM cell line. Unlike Oli-neu (De Vries and Boullerne, 2010), Oli-neuM cells, by constitutively expressing MyRF gene, can differentiate till they engage and myelinate synthetic axons upon promyelinating drug treatment (Porcu et al., 2015; Nocita et al., 2019).

**Figure 1:**
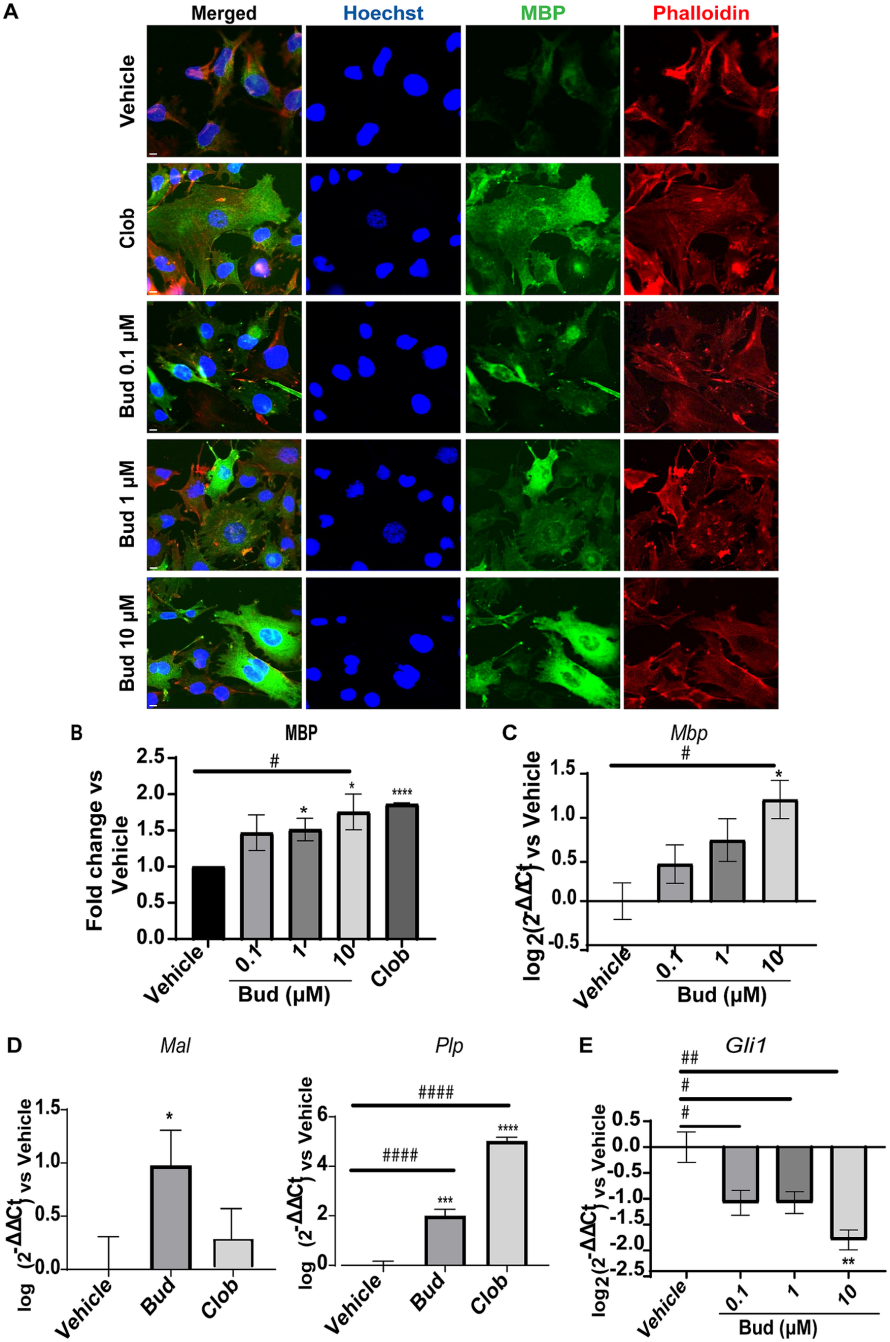
Budesonide promotes Oli-neuM differentiation and *Gli1* downregulation. (A) IF dose–response analysis of MBP levels under treatment with Budesonide (0.1; 1; 10 μM), Clobetasol (10 μM) or vehicle (DMSO 0.5%) for 48 h. Treatments were performed in differentiation medium (DM) as described in the text. ⍺-MBP primary antibody and secondary ⍺-mouse Alexa 488 (green) were used to detect MBP. F-actin was stained with Phalloidin (red); Nuclei with Hoechst (blue). Scale bar = 10 μm. (B) IF data image analyses using ScanR software (Olympus v.3.0). Mean data (±SEM) of three biological replicates (*n* = 3) were plotted in the graph using GraphPad (v.7) as Fold Change *vs* Vehicle arbitrarily set to 1; (C–E) qRT-PCR analyses of mRNA obtained from cell extracts of indicated treatments. Bud = Budesonide, Clob = Clobetasol. When not indicated the drug concentration was 10 μM. The mean data (± SEM) *n* ≥ 3 were plotted as log_2_ (2^-ΔΔCt^) versus vehicle as shown in the text. Statistical significance was analysed vs. vehicle using the two-tailed t-Student Test: *p*-value * < 0.05, ** < 0.01, **** < 0.0001; or Ordinary one-way ANOVA Tukey’s multiple comparisons test: adjusted *p*-value # < 0.05; ## < 0.01 ### < 0.001 #### < 0.0001.

We first established the effects of Budesonide on MBP expression in dose–response experiments, starting from the minimal effective concentration we have previously established in Oli-neuM cells (Porcu et al., 2015). Therefore, we tested Budesonide at concentrations of 0.1, 1 or 10 μM, using 10 μM Clobetasol as positive control or vehicle (max 0.5% DMSO) as negative control. Treatments were applied in differentiation medium (DM) for 48 h, after which the cells were processed for IF microscopy (Figures 1A,B) or qPCR analysis (Figures 1C–E). A clear dose–response relationship was observed in both IF and qPCR experiments for MBP (Figures 1B,C). To further validate these findings, we also analyzed the expression levels of the tetraspan myelin proteolipid protein (PLP), one of the most abundant proteins of compact myelin in the CNS, as well as of the myelin and lymphocyte protein MAL, a conserved tetraspan proteolipid component of both peripheral nervous system (PNS) and CNS myelin. Both Plp and Mal showed a significant increase in their expression compared to the vehicle upon 48 h of treatment with 10 μM Budesonide (Figure 1D).

To determine if Budesonide stimulates or inhibits Smo activity under our experimental condition we performed a dose–response experiment to measure Gli*1* expression levels by qPCR (Figure 1E). As expected, Budesonide acts as an inhibitor of Smo activity: *Gli1* is downregulated by Budesonide in a dose-dependent manner. Being 10 μM Budesonide the maximal Gli1 inhibition dose observed.

These results confirm that Budesonide treatment inhibits Smo activity while simultaneously promoting Oli-neuM differentiation and myelin gene expression.

**Figure 2:**
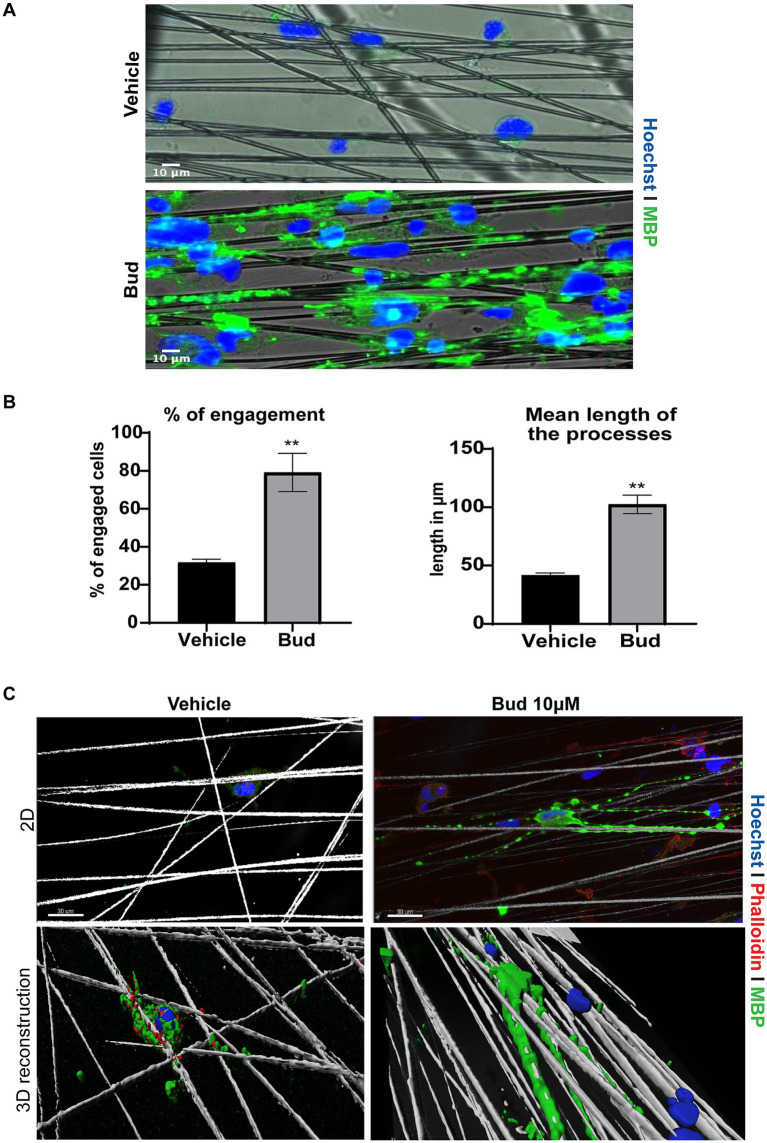
Budesonide promotes the engagement of Oli-neuM onto PS microfibers. (A) Representative IF images of Oli-neuM cells treated with 10 μM Budesonide for 72 h, grown in chambers containing aligned PS microfibers of 4 μm, mimicking axons. Images were acquired with Leica DM1600B inverted epifluorescence microscope with a 20X objective. After fixation cells were stained with anti-MBP primary and Alexa 488 secondary antibodies (FITC). Hoechst (DAPI) = nuclei. Scale bar = 10 μm. (B) *Left panel*: quantification of Oli-neuM cell engagement on PS fibers: 75 images for each treatment (*n* = 3) were acquired. The percentage (%) of engaged cells was estimated by counting the nuclei that were either on or in contact with the fibers, compared to the total number of nuclei counted within 86 μm from the fiber, as described in Del Giovane et al. (2022). The mean % (±SEM) of engaged cells of three biological replicates (3n) was plotted on the graph. *Right panel*: quantification of the mean length of the processes extending onto PS fibers. 100 engaged cells per treatment were randomly selected for this analysis. The length of the membrane processes extended along each fiber was measured (μm) using the “analyze/measure” tool of ImageJ. The means membrane length (μm ± SEM) values of three biological replicates were plotted in the graph. (C) Representative confocal images and 3D reconstruction of a typical Oli-neuM cell engaging PS fibers Panels 2D: confocal images of indicated samples. Images were acquired with 40X objective; Cells were labelled with anti-MBP Ab (FITC) and cytoskeleton was visualized with phalloidin staining (TRITC); Hoechst (DAPI) = nuclei. Scale bar = 30 μm. 3D reconstruction of the images in the upper panels is shown. 3D surface reconstruction was performed using Imaris software (Bitplane AG, Zúrich-Switzerland). The image was cropped around the cell and tilted of about 30 degrees to better visualize membrane surrounding fibers.

To determine if Budesonide treatment could induce OPCs differentiation till axon engagement (Lee et al., 2012) we cultured Oli-neuM cells in chambers containing parallel electrospun aligned PS microfibers with a 4 μm diameter (here following named “synthetic axons”), as we previously described (Del Giovane et al., 2022). After 72 h of treatment with 10 μM Budesonide or vehicle in DM media, Oli-neuM cells were fixed and processed for IF confocal microscopy analyses (Figure 2). We observed that vehicle-treated cells poorly engaged the PS fibers compared to Budesonide-treated cells, although they were plated at the same concentration and treated in parallel experiments. Indeed, in this and previous experiments, we observed that a number of them, not engaging fibers, spontaneously undergo apoptosis. On the contrary, those cells engaging synthetic axons appear stable and extend the processes along the fibers expressing MBP (Figure 2A). To estimate the number of cells engaging the fibers (Figure 2B), 75 images were acquired randomly for each sample in each of the three biological replicates (3n). Cells were counted within 86 μm from the center of the nearby fiber. Those with the nuclei attached or on the PS fiber were considered engaged, the others not engaged (Figure 2B left panel). We also quantified the length of the membrane processes, expressing MBP, that extend onto fibers. The lengthening of the membrane over the fiber was expressed in micrometres and plotted on the graph as the mean value (±SEM) of three biological replicates (Figure 2B right panel). Both the percentage of engaging cells and the length of processes extending onto the PS fibers were significantly increased by Budesonide treatment.

Altogether these data conclusively show that Budesonide rapidly promotes Oli-neuM differentiation within 72 h of treatment and increases the capacity of Oli-neuM to contact axons and effectively wrap them as shown in the 3D reconstruction (Figure 2C) obtained using the surface tool of Imaris software, allowing to visualize the cell surface of a typical cell treated with vehicle or Budesonide, wrapping the PS fibers with their processes.

### Budesonide requires Smo receptor to promote MBP expression

Since Budesonide binds to Smo CRD but also regulates glucocorticoid receptor (GR), we wished to clarify the contribution of each to MBP expression. Toward this aim, we investigated the impact of Smo on Budesonide-induced MBP expression by utilizing two isogenic cell lines, the Oli-neuM^shSmo^ and the Oli-neuM^shCTL^, previously created by infection with Lentivirus particles carrying, respectively, the pLKO.1 vector expressing Smo shRNA (shSmo) and a pLKO.1 vector expressing a control shRNA (shCTL) (Del Giovane et al., 2022).

**Figure 3:**
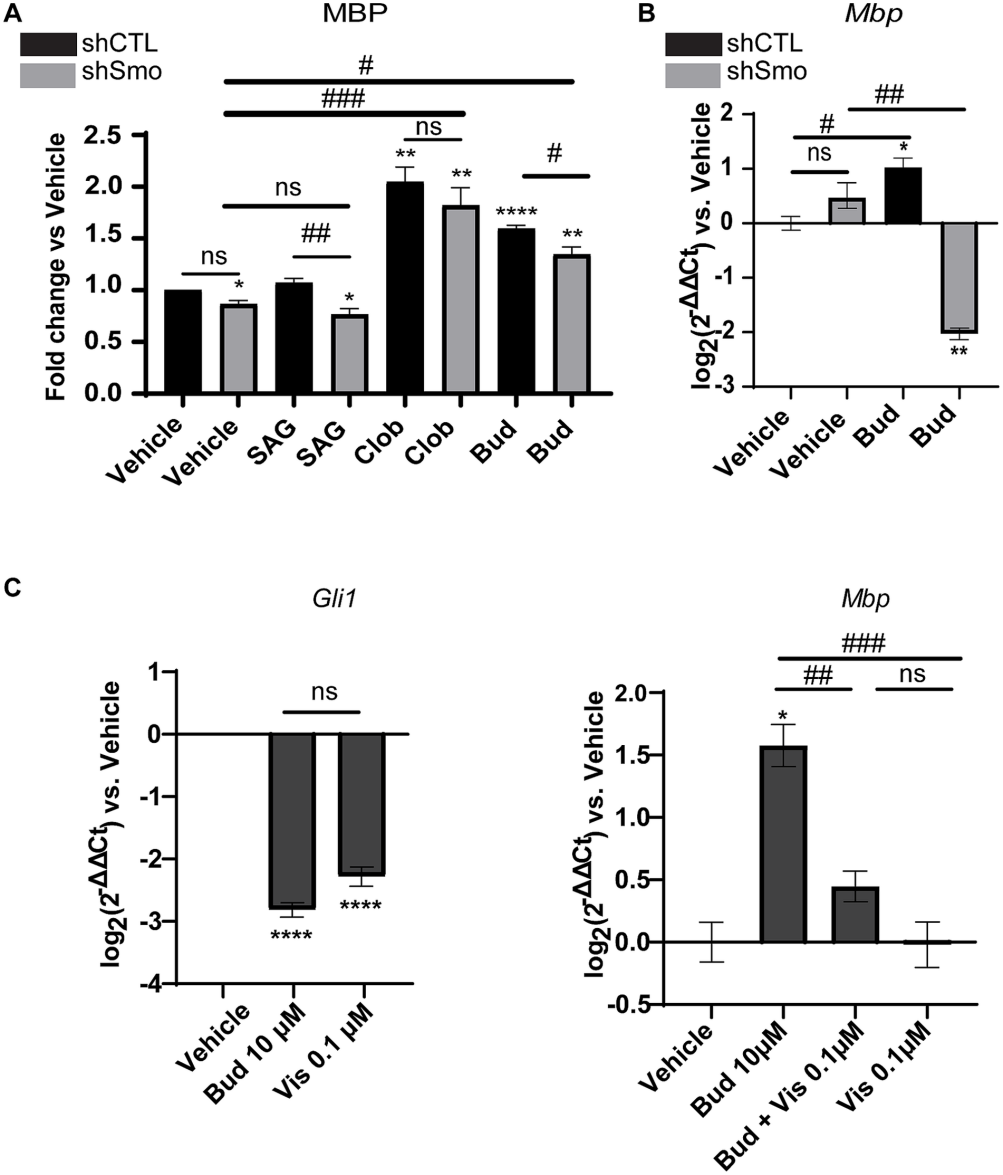
MBP expression under Budesonide treatment requires Smoothened activity. (A) IF analysis of mean MBP levels in the Oli-neuM^shSMO^ and Oli-neuM^shCTL^ cell lines under treatment with 5 μM SAG, 10 μM Clobetasol, 10 μM Budesonide or vehicle (DMSO <0.5%) in DM medium for 48 h. After fixation anti-MBP Ab and secondary anti-mouse Alexa 488 were used to stain MBP; Phalloidin staining was used to detect F-Actin; Hoechst to stain nuclei. ScanR software (3.0 Olympus) was used for image visualization and data analyses. The mean intensity data (n3 ± SEM) were plotted and statistically analyzed using GraphPad Prism version 7.00 (GraphPad Software, Inc.). Statistical analyses: to compare treatments vs. vehicle two-tailed t-Student Test was used. Ordinary one-way ANOVA followed by Dunnett’s multiple comparisons test was used to compare ShSMO treatment vs.ShSMO vehicle. One-way Anova followed by Turkey’s multiple comparison was used to compare shSMO vs. ShCTL. t-Student Test *p*-value * < 0.05, ** < 0.01, *** < 0.001, **** < 0.0001. ANOVA p-value # < 0.05; ## < 0.01; ### < 0.001. ns = non-significant (*p*-value>0.05). (B) Analyses of *Mbp* gene expression in Oli-neuM^shSMO^ and Oli-neuM^shCTL^ cell lines by qPCR after 48 h treatment with 10 μM Budesonide or vehicle (DMSO<0.5%) in DM. (C) Left panel: Analyses of Smo activity under Budesonide 10 μM (Bud) or 0.1 μM Vismodegib treatment in Oli-neuM cells. Gli1 was used as a read-out of Smo activity. Gli1 gene expression was determined by qPCR. Mean expression data were plotted on the graph as log_2_ (2^-ΔΔCt^) versus vehicle ± SEM (n ≥ 3) using GraphPad Prism version 7.00 (GraphPad Software, Inc.). Right panel MBP gene expression in Oli-neuM cells under the indicated treatments. qPCR and mean data analyses were performed as indicated in the text. Statistical analyses: to compare treatments vs. vehicle two-tailed t-Student Test was used. Ordinary one-way ANOVA followed by Turkey’s multiple comparisons test was used to compare treatments among each other. t-Student Test *p*-value * < 0.05, ** < 0.01, *** < 0.001, **** < 0.0001. ANOVA *p*-value # < 0.05; ## < 0.01; ### < 0.001. ns = non-significant (*p*-value>0.05).

Oli-neuM^shSmo^ and the Oli-neuM^shCTL^ cells were treated with 10 μM Budesonide, 5 μM SAG, 10 μM Clobetasol or vehicle (DMSO<0.5%) in DM media for 48 h and processed in parallel for quantitative automated IF microscope analysis as described in Materials and Methods. 10 μM Clobetasol or SAG 5 μM were used as a positive or negative control, respectively. Clobetasol treatment is known to induce MBP by binding GR while the second stimulates Gli1 but not MBP in Oli-neuM (Del Giovane et al., 2022). Images were analyzed and the mean data of three biological replicates were plotted in the graph and statistically analyzed (Figure 3A and Supplementary Figure S2).

Results showed that both Clobetasol and Budesonide treatments upregulate MBP protein levels in Oli-neuM^shCTL^and Oli-neuM^shSmo^ cells. However, Budesonide activity is significantly reduced in Oli-neuM^shSmo^ treated cells compared to Oli-neuM^shCTL^ indicating that Smo gene silencing markedly reduces MBP levels in Budesonide-treated cells. This effect is below significance in the presence of Clobetasol or vehicle treatment (Figure 3A). To corroborate these data, we have analyzed *Mbp* gene expression in Oli-neuM^shCTL^ and Oli-neuM^shSmo^ cells upon Budesonide and vehicle treatments (Figure 3B). We observed a striking reduction of *Mbp* mRNA in Oli-neuM^shSmo^ compared to Oli-neuM^shCTL^ Budesonide treated cells while the vehicle did not show significant differences (Figure 3B). These data suggest that MBP mRNA is more sensitive to treatment than MBP protein levels. This difference could result from the distinct turnover rates of the mRNA and protein.

To confirm the capacity of Budesonide to act as an inhibitor of Smo activity in our experimental conditions we analyzed the effect on *Gli1* gene expression under Budesonide compared to Vismodegib treatments in Oli-neuM. Vismodegib is a well-characterized competitive antagonist of Smo activity and it binds to the receptor orthosteric site within the TMD bundle (Byrne et al., 2016; Huang et al., 2018). In preliminary experiments, we observed that the minimal effective Gli1 inhibitory dose in qPCR of Vismodegib is 0.1 μM (Figure 3C). Compared to 0.1 μM Vismodegib, 10 μM Budesonide has similar inhibitory activity on Gli1 gene expression (Figure 3C left panel).

**Figure 4:**
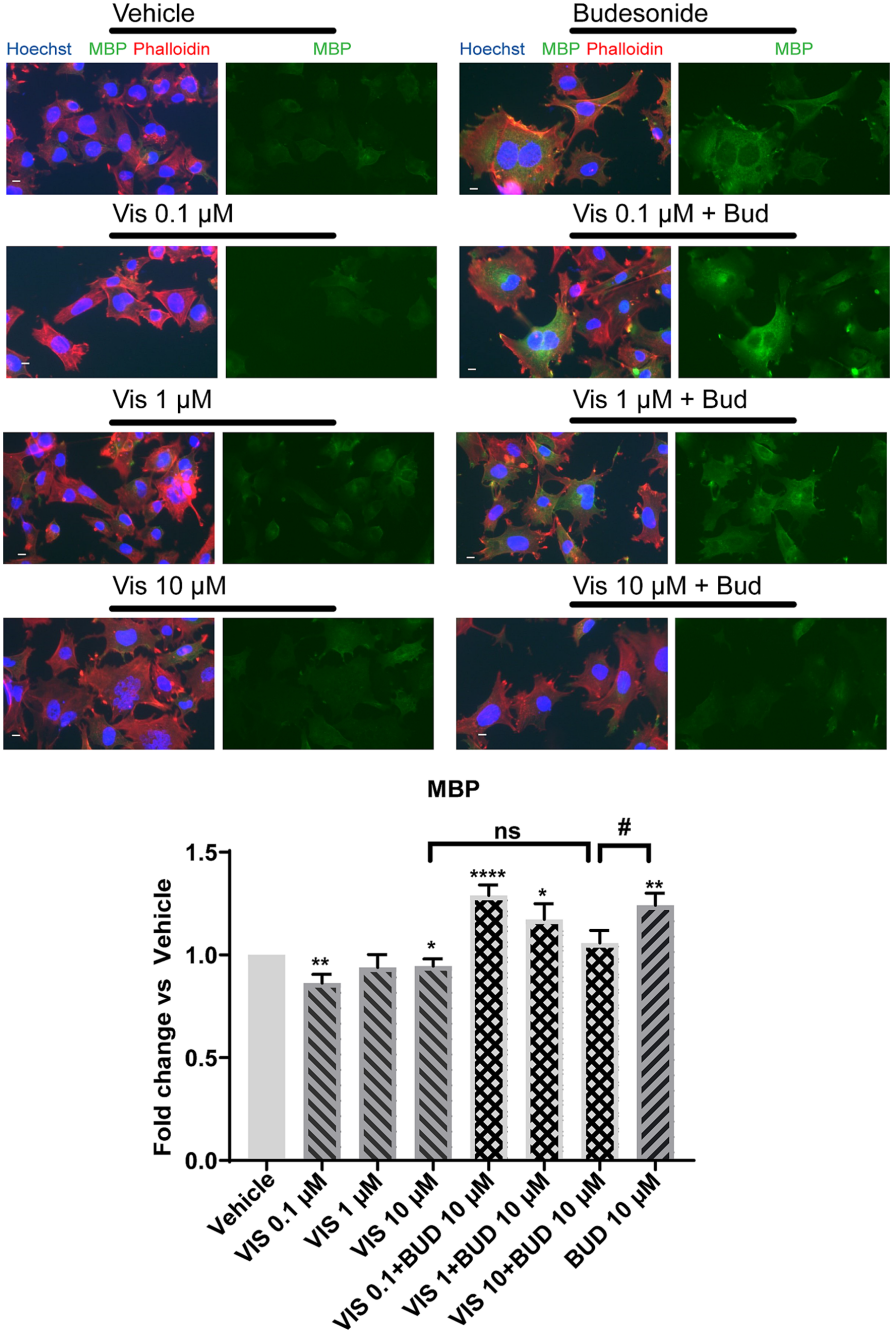
Vismodegib co-treatment with 10 μM Budesonide reduces MBP cytosolic levels in a dose dependent manner. Representative images of IF analysis of the mean MBP levels (±SEM, *n* = 3) in Oli-neuM cell line under treatment with 0.1, 1, 10 μM Vismodegib alone or in combination with 10 μM Budesonide. After fixation anti-MBP Ab (FITC), Phalloidin (TRITC), Hoechst (DAPI) were used to detect MBP, F-Actin and Nuclei, respectively. ScanR software (3.0, Olympus) was used for image visualization and mean data analyses. Mean data were statistically analyzed using GraphPad Prism (*vs* 7.00, GraphPad Software, Inc.). Statistical analysis *vs* vehicle was performed using the two-tailed t-Student Test: *p*-value * < 0.05, ** < 0.01, **** < 0.0001; or Ordinary two-way ANOVA Turkey’s multiple comparison test: *p*-value # <0.05. ns = non-significant.

We then wondered if the combined use of two Smo inhibitors, namely 0.1 μM Vismodegib and 10 μM Budesonide, could have combinatorial effects on MBP expression (Figure 3C right panel). Indeed, the combined use of 10 μM Budesonide and 0.1 μM Vismodegib reduces *Mbp* gene expression compared to 10 μM Budesonide alone indicating that they partially obstacle each other action. To confirm that this effect is reflected also on the MBP cytosolic levels we determined by IF analyses the effects on MBP levels of the combined use of 10 μM Budesonide with dose increase of Vismodegib (0.1, 1, or 10 μM; Figure 4). Clearly, at the increase of Vismodegib concentration, the efficacy of Budesonide to promote MBP increase diminishes. This latter result supports the view that Vismodegib by binding to the Smo TMD might contribute to reduce Budesonide access to the CRD, thereby reducing its efficacy in stimulating MBP.

We concluded that Budesonide acts as an inhibitor of Smo canonical pathway in Oli-neuM and through Smo promotes MBP gene expression (Figure 3) and consequently an increase in the MBP cytosolic levels (Figures 3, 4).

### Budesonide promotes MBP expression via LKB1/AMPK signaling

The molecular signaling through which Budesonide treatment stimulates MBP gene expression via Smo binding remained to be clarified.

In a previous study, we demonstrated that the quinolone GSA-10 activates MBP gene expression through AMPK activation (Del Giovane et al., 2022). Additionally, various ligands can activate non-canonical pathways by binding to Smo, resulting in AMPK activation in response to changes in nutrient intake or environmental conditions (Akhshi et al., 2022). Therefore, we hypothesized that also Budesonide might trigger this pathway to promote AMPK phosphorylation.

AMPK is a kinase involved in various intracellular processes and cytoskeleton rearrangement (Neumann et al., 2019). It is important to note that Gli1 is a known target of activated AMPK (Teperino et al., 2012). When activated, AMPK phosphorylates Gli1 at serine/threonine residues (Ser102, Ser408 and Thr1074), decreasing both its transcriptional activity and protein stability (Li et al., 2015; Di Magno et al., 2016). Conversely, it increases Gli1 cytoplasmic localization and its interaction with the E3 ubiquitin ligase *β*-TrCP, leading to Gli1 degradation by the proteasome (Zhang et al., 2017).

**Figure 5:**
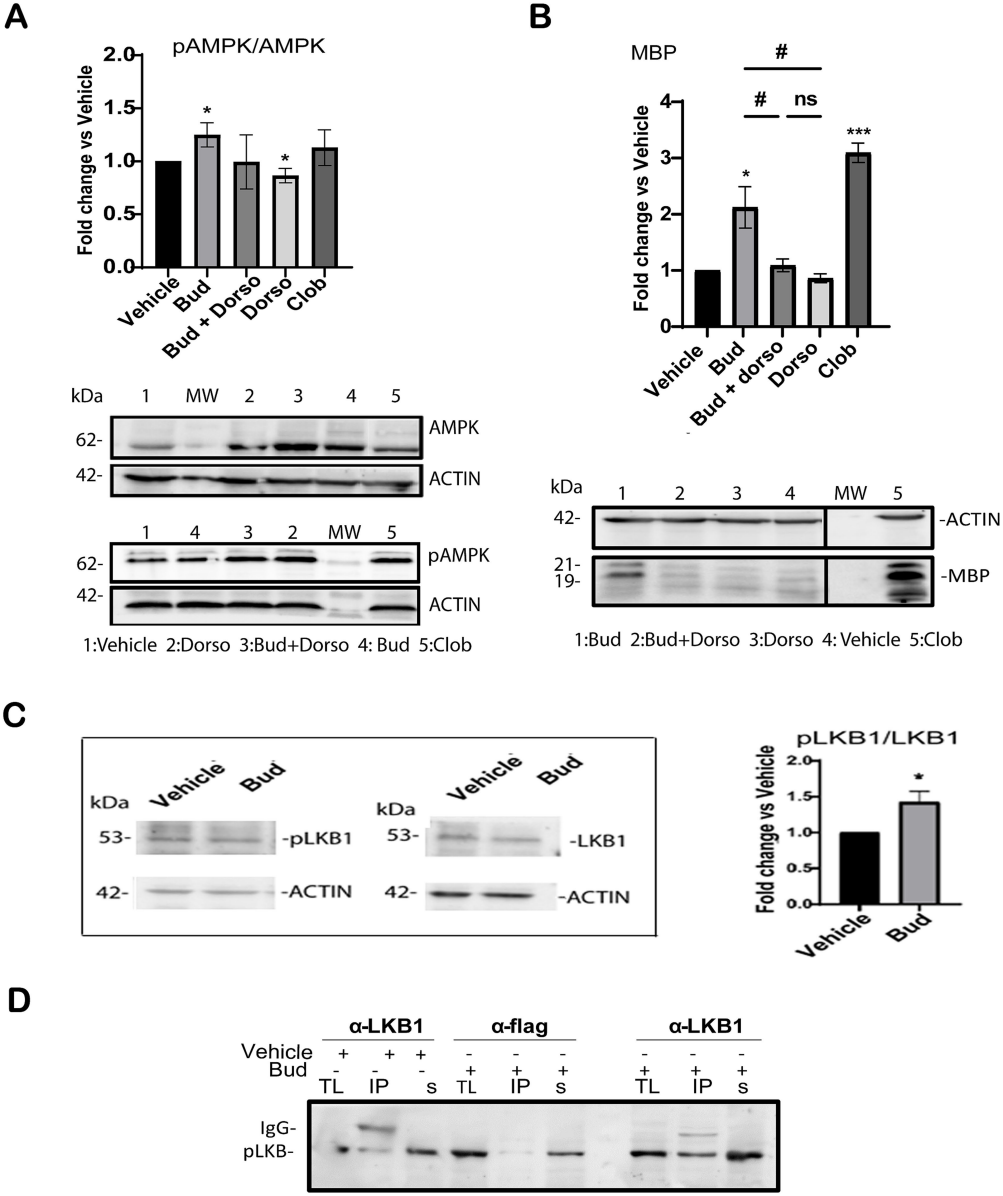
Budesonide stimulates MBP expression via LKB1/AMK signaling. Immunoblot analyses of total lysates extracts from Oli-neuM cells treated for 48 h with the indicated compound as specified in the text. IB analyses of lysates with anti-AMPK (AMPK), anti-phosphorylated AMPK Ab (phoAMPK) **(A)**; with anti-MBP (MBP) **(B)**. After treatment and IB, bands were quantified by ImageJ 1.54d and data were plotted in the graph. Left panel quantification of IB data (3n ± SEM) using GraphPad Prism version 7.00 (GraphPad Software, Inc.). Right panel: Typical IB images for this experiment. MW: molecular weight; Dorso = Dorsomorphin; Bud+Dorso = Budesonide+Dorsomorphin; Clob = Clobetasol; vehicle = DM + 0.5%DMSO. Protein levels were normalized to vehicle control, arbitrarily set to 1. **(C)** Immunoblotting (IB) analysis of total lysates detected with anti-pLKB or anti-LKB after indicated treatment. Bands were quantified using Image J software. Right panel: data were plotted on the graph using GraphPad Prism Software. **(D)** Immunoprecipitation of LKB1. Treatment, lysates extraction and IP reactions (IP) were performed as indicated in the text. Anti-LKB1 (IPαLKB1) and anti-Flag Ab (IPαFLAG) were used for IP reactions. Anti-phosphorylated LKB1 (p-LKB1) was used for detection. IgG position and MW marker bands are indicated. + = addition; − = absence of treatment. TL = total lysate input; IP = immunoprecipitated pellet; s = IP supernatant; Statistical significance was analyzed using the two-tailed t-Student Test: *p*-value * < 0.05, **** < 0.0001; One-way ANOVA followed by multiple comparisons test was used between treatments (adjusted *p*-value # < 0.05); ns = not-significant (adjusted *p*-value>0.05).

Therefore, we analyzed the effects of Budesonide treatment alone or in combination with the selective phospho-AMPK inhibitor Dorsomorphin (DRS) (Lo et al., 2019; Sun et al., 2021) on AMPK activation and MBP expression. 3 μM DRS either alone or in combination with 10 μM Budesonide was used to treat the cells for 48 h. After protein extraction, AMPK activity was determined by Immunoblotting (IB) analyses (Figure 5A). The results revealed that 48 h of Budesonide treatment induces AMPK phosphorylation, while the combined use of DRS substantially reduces phospho-AMPK levels to those observed in control conditions (Figure 5A) and MBP expression (Figure 5B). These data confirmed that 10 μM Budesonide treatment promotes AMPK phosphorylation after 48 h treatment and this signal is required for MBP expression.

In their 2012 study, Teperino et al. (2012) demonstrated that LKB1 can serve as an upstream regulator of AMPK phosphorylation via Smo. Therefore, we analyzed the effects of 10 μM Budesonide treatment on LKB1 activation in total lysates of Oli-neuM. Immunoblot quantification of the mean relative amounts of pLKB and LKB present in the total extracts (*n* = 3) reveals a significant increase of LKB phosphorylation in Budesonide-treated cells compared to vehicle (Figure 5C). To better visualize activated LKB1 we immunoprecipitated the LKB1 protein from total lysates of Oli-neuM cells treated as above (Figure 5D). Anti-LKB1 Ab (IP_αLKB1_) or, as a mock reaction, with anti-FLAG Ab (IP_αFLAG_) was used to IP the LKB1 protein. IP pellets and Supernatants were then processed for IB analyses using anti-PhoLKB1and anti-LKB1 Ab in parallel experiments (Figure 5D). Clearly, the IP_αLKB1_ pellets of Budesonide-treated cells contained more phosphorylated LKB1 compared to vehicle-treated IP_αLKB1_ pellets (Figure 5D). This result is specific as no phosphorylated LKB1 could be pelleted in the mock IP reaction (IP_αFLAG_).

Altogether, the results show that Budesonide promotes MBP expression via a signaling that requires AMPK phosphorylation. Given the known role of LKB1 to act downstream of Smo and upstream of AMPK our data support the view that Budesonide treatment stimulates both kinase phosphorylation on its way to promote MBP expression.

### Inhibition of GR activity reduced MBP protein levels but did not affect MBP gene expression under budesonide treatment

**Figure 6:**
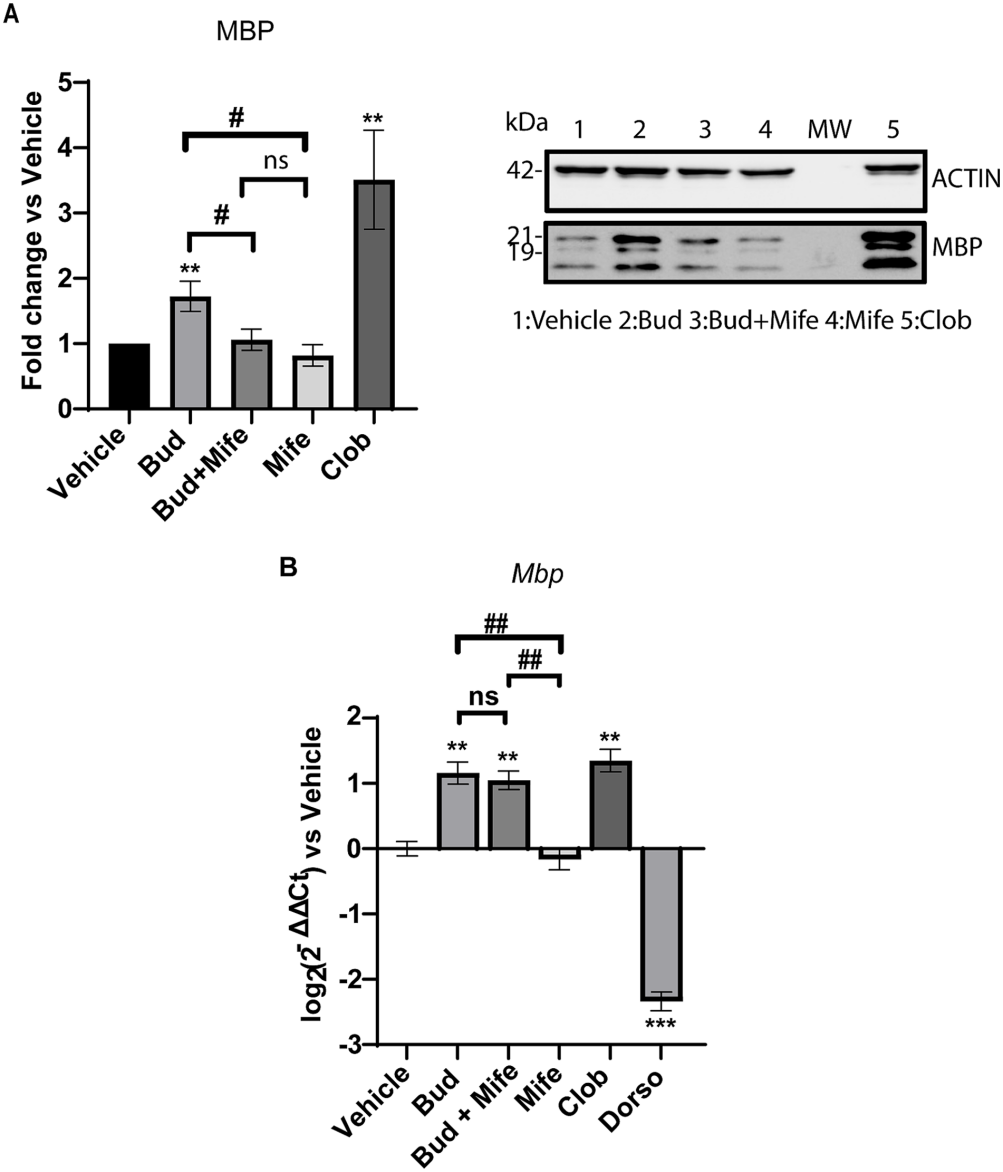
Inhibition of GR activity using Mifepristone reduces MBP cytosolic levels but not MBP gene expression. (A) Immunoblotting analyses under GR inhibitor Mifepristone. Oli-neuM were treated as indicated in the text and extracts were processed for IB analyses. MBP was detected using anti-MBP Ab and bands were quantified using as an anti-βActin Ab as internal control. Bud = Budesonide; Mife = Mifepristone; Clob = Clobetasol; Dorso = Dorsomorphin. (B) MBP gene expression was estimated using qRT-PCR. Treatment and cDNA were obtained from Oli-neuM cells treated as indicated in the text. Data are the mean of 3n replicates and are plotted using GraphPad Prism version 7.00 (GraphPad Software, Inc.). Protein Data are reported as mean increase over vehicle, arbitrarily set to 1. qRT-PCR data are reported as the log_2_(2^-ΔΔC^) mean fold induction over vehicle set to 0. Statistical significance was analyzed vs. vehicle using the two-tailed t-Student: *p*-value * < 0.05, ** < 0.01, *** < 0.001 and among treatment using One-way ANOVA followed by multiple comparisons: adjusted *p*-value # < 0.05, ns = not significant (*p*-value>0.05).

Since Budesonide exerts its anti-inflammatory activity by binding to GRα subunit, we wondered if both GR and Smo receptors are involved in the stimulation of MBP gene expression. To address this, we determined the effects of Mifepristone alone or in combination with Budesonide in Oli-neuM after 48 h treatment. Mifepristone is an anti-progesterone drug that inhibits GR activity by binding to the GRα subunit (Pelaia et al., 2016; Caramori et al., 2022). In these experiments, Oli-neuM cells were plated for 24 h in GM, prior to being treated with 10 μM Budesonide, 0.1 μM Mifepristone, with both drugs or with vehicle control for 48 h in DM. Clobetasol was used as a positive control for MBP expression. Total lysates were processed for IB analysis as described in Material and Methods (Figure 6A). As expected, IB analyses showed a significant increase in MBP under Budesonide and Clobetasol treatment. No effect was observed under Mifepristone treatment compared to the vehicle. The co-treatment of Budesonide and Mifepristone (Bud+Mife) impaired the stimulatory effects of Budesonide on MBP levels (Figure 6A respective lanes). We then ascertain if Mifepristone had any effect on reducing Budesonide-mediated MBP gene transcription through qPCR (Figure 6B). We observed that while Clobetasol (Clob) and Budesonide (Bud) stimulate MBP gene expression, the combination treatment with Budesonide plus Mifepristone did not alter this pattern.

Overall, IB and qPCR data shows that Budesonide’s stimulation of MBP in Oli-neuM cells involves both GR and Smo receptors, however GR controls solely MBP protein levels but not MBP gene expression.

### Computational analysis of budesonide binding modes with the Smo CRD

To further clarify how Budesonide can antagonize Smo canonical pathway, we performed extensive computational investigations. Given the importance of Smo as anticancer target, several crystallographic structures of the receptor bound to either agonists or antagonists are available for modeling studies (Rana et al., 2013; Byrne et al., 2016; Luchetti et al., 2016; Xiao et al., 2017). These structures allow identifying two main Smo domains to which receptor’s ligands can bind: the extracellular CRD and the seven-helix domain (TMD). The CRD is essential for the localization at the primary cilium (Rana et al., 2013; Byrne et al., 2016; Zhang et al., 2021), and it interacts with two major groups of small molecules: hydroxylsteroids and GCs. TMD can interact with both agonists such as SAG and antagonists, including Cyclopamine, Vismodegib and Smoothened antagonist (SANT-1) (Byrne et al., 2016; Luchetti et al., 2016). Notably, cholesterol and oxysterols have been shown to act as endogenous activators of Smo by engaging the extracellular CRD and, in turn, to positively regulate Smo/Gli1 signaling (Luchetti et al., 2016).

Despite the well-known capability of Budesonide and of other GCs to bind the CRD domain of hSmo, the precise structural details of this ligand-receptor interaction remain unclear (Rana et al., 2013). To date, only one structural study has been conducted by Huang et al. (2016), who used Nuclear Magnetic Resonance (NMR) experiments to outline the binding region of this corticosteroid in the CRD domain of both human and Drosophila Smo; however, the atomistic model of the binary complex was only released for the Drosophila variant. To get more insights on how GCs binding to Smo CRD can result in the inhibition of Smo/Gli1 signaling while promoting AMPK activation and MBP expression and to elucidate the molecular determinants of the Budesonide-hSmo interaction, we analyzed Budesonide’s binding mode within the hSmo CRD *in silico*.

Initially, molecular docking calculations were conducted using Glide 9.3 software and employing as a target the experimental 3D structure of hSmo in complex with cholesterol bound at CRD, due to its chemical similarity with Budesonide (see Materials and Methods for details). The lowest-energy and most recurring docking pose shows Budesonide occupying the same lipophilic cleft as cholesterol, delineated by residues L112, I156, V157, I496, and L489 (Supplementary Figure S5).

**Figure 7:**
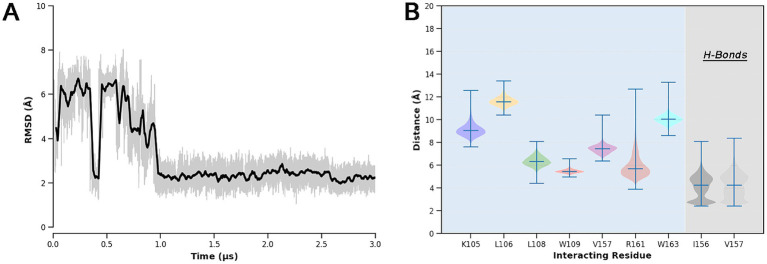
(A) RMSD of the Budesonide’s heavy atoms over the MD trajectory with respect to the starting docking pose. The bolded line represents a moving average computed with a rolling window of 3 ns; actual RMSD fluctuations are shown in slight transparency. (B) Violin plots of the most relevant BUD-hSmo interactions. Lipophilic contacts were computed as distances of the centers of mass of Budesonide and of the side chains of the single residue involved.

**Figure 8:**
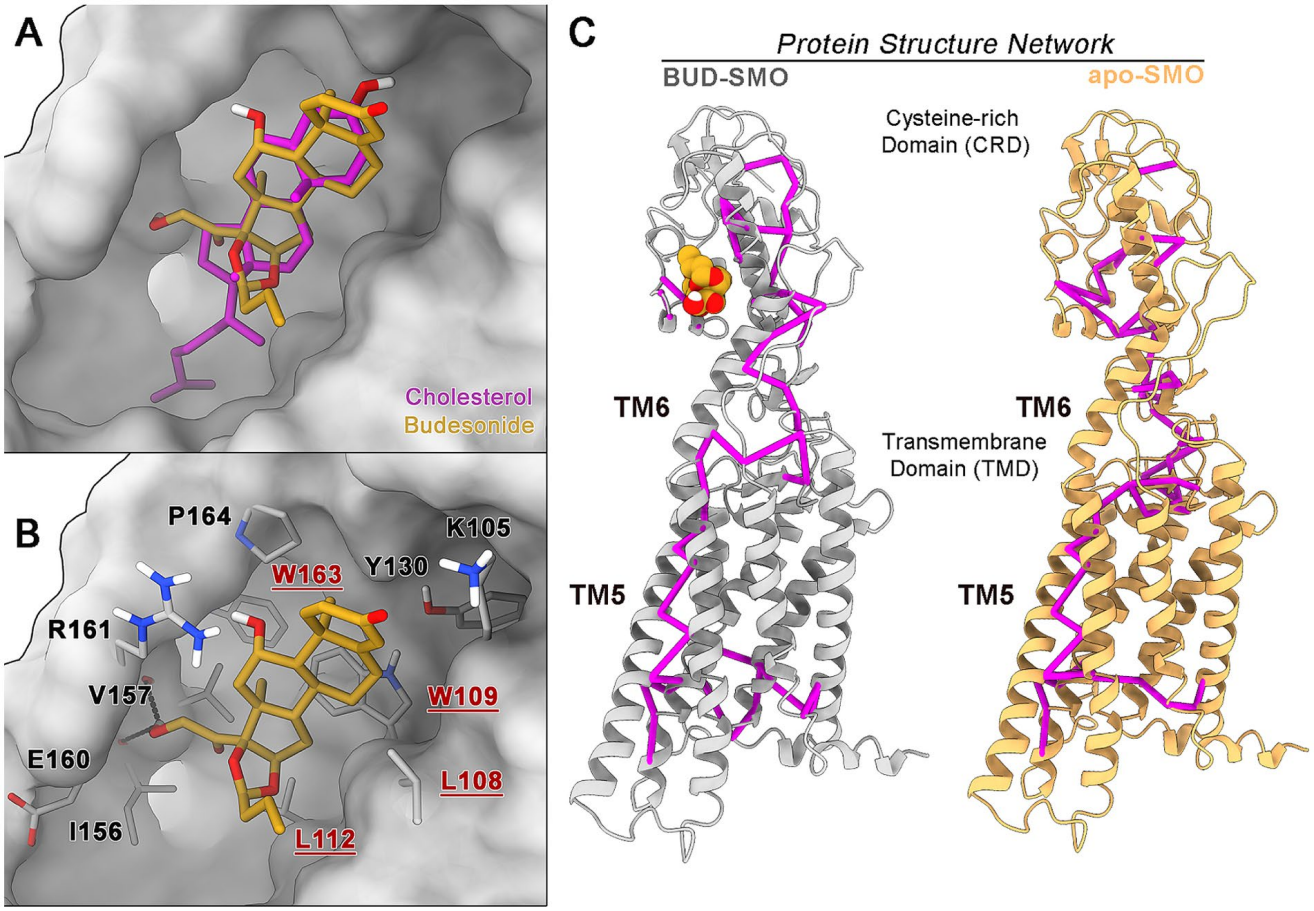
(A) Superposition between the average MD pose of Budesonide with the experimental structure of cholesterol-hSmo (PDB code: 5L7D). (B) MD-predicted binding pose of Budesonide at the hSmo CRD. The protein is depicted as a silver surface; Budesonide, cholesterol and the interacting residues are highlighted in orange, magenta and grey sticks (red labels refer to amino acids experimentally found to interact with Budesonide, Huang et al., 2016), respectively. (C) Protein structure network (PSN) computed for the BUD-hSmo complex and apo-Smo. The most relevant metapaths are shown as magenta edges.

Then, to better evaluate the energetics of the predicted binding mode and account for solvation and protein flexibility effects, extensive MD simulations (3 μs long) were performed on the BUD-hSmo docking complex. The analysis of the ligand RMSD over time revealed a rearrangement during the initial microsecond of simulation, after which Budesonide reaches a conformation conserved for the rest of the simulated timescale (»2 μs) (Figure 7). Interestingly, the final binding mode of Budesonide closely resembles that of cholesterol in experimental complexes (Figures 8A,B). In detail, the pose is stabilized by a set of lipophilic contacts between the ligand’s steroid nucleus and the sidechains of L108, W109, L112, Y130, I156, V157, and P164, as well as by two hydrogen bonds detected between Budesonide’s 21-hydroxyl group and the backbone carbonyl moieties of I156 and V157 (Figures 7B, 8B). Notably, the predicted binding model aligns well with the available experimental data on the BUD-hSmo interaction. As shown in Figure 8, the ligand is indeed surrounded by the amino acids (L108, W109, G111, L112, G162 and W163) that exhibited the most significant chemical shift changes in the NMR experiments performed by Huang et al. (2016).

To investigate the relationship between the Budesonide’s binding mode and its pharmacological activity, a second MD simulation was performed on the apo-hSmo receptor to compare how Budesonide binding can affect the GPCR dynamics relative to its basal unliganded state. We particularly focused on the allosteric networks known to govern GPCR functioning by using a graph-based approach, namely protein structure network analysis (PSN) (see Materials and Methods for details), which identifies the strongest inter-residue interactions along an MD trajectory (hereafter referred to as to metapath). In both the BUD-hSmo system and the apo receptor, PSN detected long-range communications between the Budesonide/Cholesterol binding pocket in the CRD and the intracellular termini of TM5 and TM6, in line with the established regulatory role of the CRD in the activation process of Smo (Huang et al., 2018). However, interesting differences were observed in the receptor areas touched by the identified communication networks. Specifically, in the presence of Budesonide, the metapath shows a more direct involvement of the upper portions of TM5 and TM6, with a series of communication hubs (residues D473, Q477, W480, Y487, and Q491) detected exclusively under these conditions. Conversely, in the apo receptor, the interdomain edges primarily involve residues from the ECL1 and ECL2 loops.

**Figure 9:**
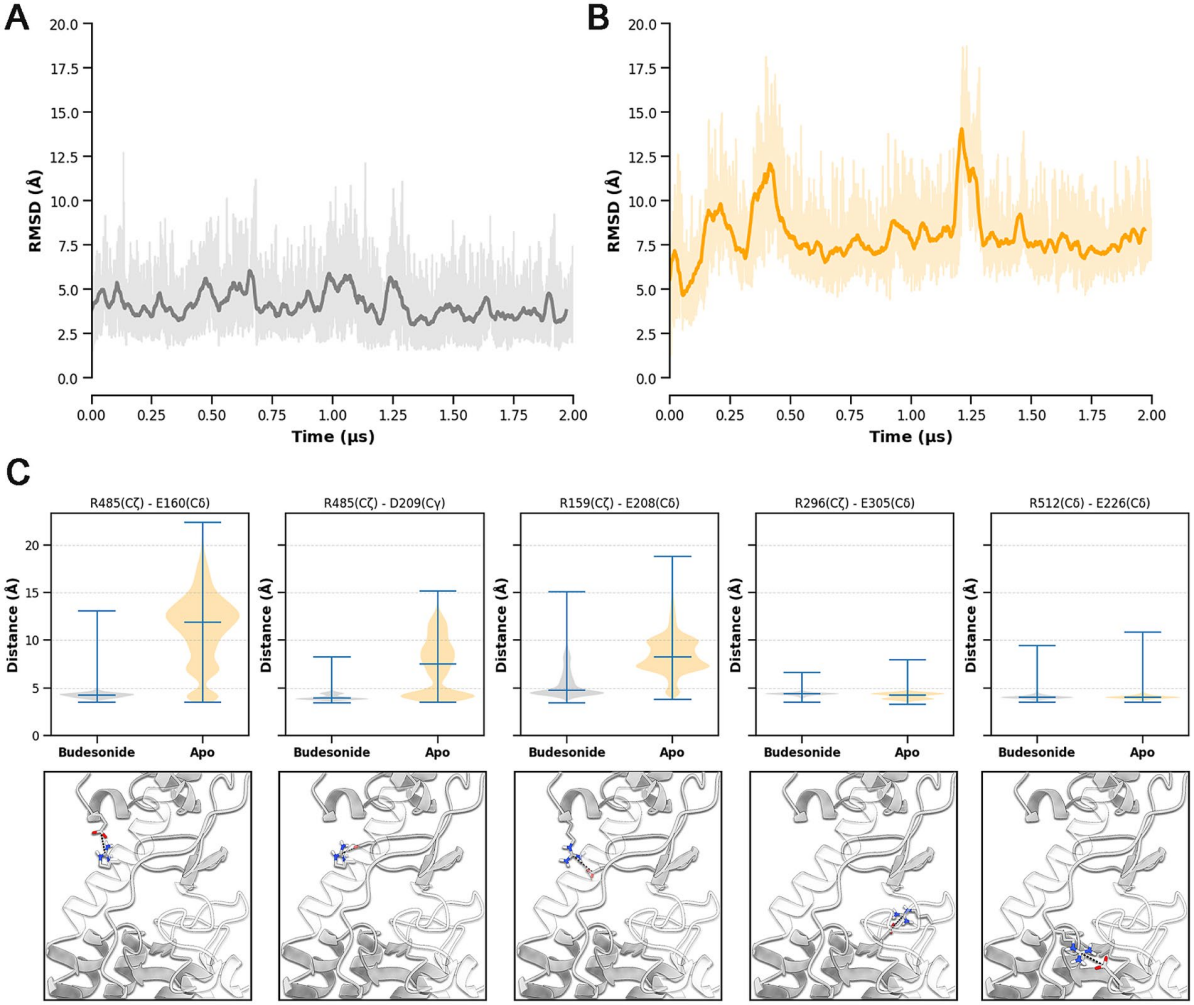
RMSD of the CRD computed for the BUD-hSmo (A) and apo-hSmo (B) systems. Prior to RMSD calculation, a differential alignment on the TMD (Cα atoms) was performed to optimize the detection of the interdomain movements. The bolded line represents a moving average computed with a rolling window of 3 ns; actual RMSD fluctuations are shown in slight transparency. (C) Violin plots and 3D representation of the TMD/linker domain-CRD salt-bridges detected over the MD trajectories.

In parallel, the distinct behavior of the receptor in the two systems was also highlighted by the analysis of CRD movements over the MD trajectories. As evidenced by the RMSD plots shown in Figure 9A, Budesonide appears to reduce the fluctuations of the CRD relative to the TMD compared to the apo receptor (Figure 9B). At an atomistic level, this stabilization is due to a more stable network of electrostatic interactions in the BUD-hSmo system, connecting the top part of the TMD and linker domains with the CRD. In line with previous computational studies (Bansal et al., 2023), we identified five salt bridges stabilizing this interdomains connection: R485-E160, R485-D209, R159-E208, R296-E305 and R512-E226 (Figure 9B). Indeed, while these contacts were maintained for most of the simulation in the BUD-hSmo trajectory, three of them (R485-E160, R485-D209, R159-E208) were gradually lost in the apo receptor, explaining the greater RMSD fluctuation observed in absence of the ligand (Figure 9B).

Altogether, our results suggest that Budesonide exerts its unique regulatory activity on Smo by promoting the compaction of the CRD domain onto the TMD and enhancing the allosteric communication between the CRD and helices TM5 and TM6. These findings, along with the detailed atomistic model of the Budesonide-hSmo interaction, may have significant implications in drug discovery, potentially guiding the design and development of new compounds with promyelinating properties like those of Budesonide.

## Discussion

The mechanism by which endogenous ligands regulate Smo, directing canonical or non-canonical signaling in oligodendrocytes, has been the subject of ongoing debate (Akhshi et al., 2022; Fang et al., 2022). Here we show that targeting the CRD of the Smo receptor with Budesonide is a promising and sustainable strategy to modulate the Hh signaling pathway toward myelination *in vitro* and we give a mechanistic view of how these events could occur.

Over the past few years, GCs binding to Smo have emerged as potent drugs promoting myelination in various studies (Najm et al., 2015; Porcu et al., 2015; Del Giovane et al., 2022; Fang et al., 2022). However, the specific mechanisms behind their action remain unclear (Joubert et al., 2010; Najm et al., 2015; Porcu et al., 2015). Among them, Budesonide was first identified in a phenotypical screen based on its ability to stimulate O4 marker in Oli-neu cells (Joubert et al., 2010). Later it was shown in fibroblasts to prevent the translocation of Smo to the primary cilium membrane by keeping Smo in the endosomes and thereby negatively regulating Gli1 signaling (Wang et al., 2012). These findings were significant, particularly considering current research showing that specific targeting of Smo with the quinolone GSA-10 could promote OPC differentiation in mouse models for demyelination (Del Giovane et al., 2022). Based on these data, we hypothesized that Budesonide could provide insight into how GCs binding to Smo might promote OPC differentiation through the inhibition of Gli1-mediated signaling.

In this study we investigated the role of Budesonide in promoting myelination by examining its effects on MBP gene expression and Oli-neuM to differentiate till to wrap synthetic axons. We showed that 72 h of treatment with 10 μM Budesonide significantly stimulates Oli-neuM engagement of parallel PS fibers and MBP expression. The role of Smo in myelination was investigated by studying MBP gene expression levels after silencing the Smo gene in Oli-neuM. Additionally, the role of the GR was explored by treating cells with both Budesonide and Mifepristone (RU-486), an antagonist of GR action. Our findings indicate that Budesonide promotes MBP gene expression under a signaling that requires Smo while Budesonide regulates MBP cytosolic levels mainly through GR.

Our functional studies show that the expression of the Gli1 gene is reduced when Oli-neuM cells are treated with Budesonide, confirming that OPC differentiation requires inhibition Gli1 signaling (Nocera et al., 2024). Furthermore, treating Oli-neuM cells with shSMO significantly decreases the expression of the MBP gene, confirming that MBP expression depends on Budesonide binding to Smo.

We observed that the activity of the GR is necessary for modulating cytosolic MBP levels. Even at high dosages, Mifepristone does not alter MBP gene expression but does reduce cytosolic MBP upon Budesonide co-treatment. These findings align with previous studies showing that MBP gene expression and mRNA translation in OPCs are separate processes, with transcription occurring in the nuclei and translation taking place in the cytosol in response to Fyn kinase activation (White and Krämer-Albers, 2014). The increase in cytosolic MBP levels leads to the enlargement of oligodendrocyte membrane, which is important for initiating axon engagement (Zuchero et al., 2015). Our findings suggest that Budesonide treatment can independently stimulate MBP gene transcription and translation through the regulation of Smo and GR receptors, respectively.

In our investigation of the signaling pathway from Smo inhibition by Budesonide binding to MBP gene expression, we discovered that the downregulation of Gli1 is paralleled by the phosphorylation of LKB1 and AMPK. When AMPK phosphorylation is inhibited by co-treatment with Dorsomorphin and Budesonide, MBP expression is reduced. It is not surprising to find that the non-canonical pathway activated by Smo during Budesonide treatment involves the LKB1/AMPK axis, as this signaling has been known to regulate various cellular processes (Akhshi et al., 2022). Therefore, our data support the idea that Smo inhibition by Budesonide leads to Gli1 downregulation and MBP gene expression through a signaling pathway involving Smo and passing through LKB1 and AMPK activation.

To provide mechanistic insight on how Budesonide can favor MBP gene expression and thereby myelination of synthetic axons, we performed extensive molecular dynamics studies based on the NMR of Smo structure upon inhibitors binding. The structural details of Budesonide binding to CRD domain were previously studied in Drosophila by NMR. This study clarified that the SmoCRD is an extracellular flexible domain required for Smo function in proliferation. Therefore, SmoCRD ablation in Drosophila impairs Smo signaling capacity (Rana et al., 2013). More recently, it has been shown that the CRD conformational changes induced by cholesterol binding are crucial for Hh signaling and Smo intracellular localization (Xiao et al., 2017; Hu et al., 2022). Following this path, we have built an in-silico interaction model according to which Budesonide exerts its inhibitory activity on human Smo by reducing the flexibility of the receptor CRD, which allosterically alters the behavior of the TM5 and TM6 helices, crucial for activation. In other words, Budesonide stabilizes the orientation of SmoCRD relative to the TMD. These data clarify how Budesonide binding to the CRD can interfere with endogenous ligand binding.

The idea that differential binding of ligands to the various Smo domains might regulate the outcome of Smo signaling was previously suggested by the observation that binding of different inhibitors to Smo TMD causes structural changes that inhibit cholesterol binding and tumor growth (Byrne et al., 2016; Luchetti et al., 2016; Xiao et al., 2017). In the current model, cholesterol binds to a shallow hydrophobic groove in the CRD, positioned >10 Å above the extracellular leaflet of the plasma membrane. Structure-guided mutations, which abrogate this binding, also impair Shh signaling in both cultured cells and mouse embryos (Byrne et al., 2016; Luchetti et al., 2016; Kinnebrew et al., 2022). Ptch1 transport activity is likely to regulate Smo by reducing the binding of cholesterol to the CRD, as previously suggested in other cellular systems (Xiao et al., 2017).

The data presented here provides insight into how Budesonide could promote OPC differentiation. We propose that when Budesonide binds, it reduces the flexibility of the CRD. Such an event potentially is expected to inhibit cholesterol-mediated activation of Smo (Lubetzki et al., 2020) and may result in Smo’s inability to reach the primary cilium and consequently promote Gli1 expression.

Myelin, which is enriched in cholesterol, is a major target of immune attacks in chronic neurological disorders like MS. Since cholesterol can activate Smo (Byrne et al., 2016; Luchetti et al., 2016) and thereby influence OPCs behavior, we propose that Smo might function as a “cellular rheostat” in parenchymal OPCs by sensing extracellular cholesterol levels. It is tempting to speculate that during demyelination, the excess cholesterol released from damaged myelin, by leading to Smo activation, causes Gli1 gene expression and thereby pushes OPCs toward prioritizing proliferation over differentiation. In healthy individuals, this mechanism maintains a balance between keeping parenchymal OPCs numbers stable and promoting their differentiation into OLs when needed. This balance is essential for repairing the myelin sheath in normal conditions and may be altered in MS pathology. The concept that OPCs might act as “cholesterol sensors” through Smo is supported by studies demonstrating that the genetic removal of Smo in mouse models of demyelination does not impact the percentage of OPCs differentiating into myelinating OLs (Nocera et al., 2024). In fact, according to our model, the genetic removal of Smo in OPCs would hinder their ability to ‘sense’ excess cholesterol during demyelination. This would result in the ongoing differentiation of OPCs into OLs at a steady-state level.

Importantly, the ability of Smo to act as a “rheostat” of cholesterol sensor in OPCs can be exploited pharmacologically for remyelination purposes. Following this line, we previously proved that the GSA-10, a molecule developed based on the pharmacophore of the Smo agonists SAG and Purmorphamine (Marinelli et al., 2016), exerts potent promyelinating effects both *in vitro* and *in vivo* by inhibiting Gli1 activity (Del Giovane et al., 2022). The ability of GSA-10 to modulate stem cells differentiation is not restricted to OPCs, as also mesenchymal progenitor cells, C3H10T1/2, are stimulated to differentiate in osteoblasts (Gorojankina et al., 2013; Fleury et al., 2016). Together our data indicate that regulation of Smo by regulating CRD flexibility and thereby Cholesterol binding to SMO pocket might be a general way to direct Smo toward a “non-canonical” signaling.

In conclusion, this work illuminates the molecular mechanisms by which Budesonide promotes OPCs differentiation through its inhibitory effects on Smo CRD flexibility. Moreover, supports previous evidence indicating that targeting Smo with inhibitors activating the LKB1 and AMPK signaling is a promising and sustainable strategy to modulate the Hh signaling pathway. Given that pharmacological targeting of Smo TMD has been widely used to develop anticancer agents (Byrne et al., 2016; Luchetti et al., 2016; Rimkus et al., 2016), we believe that utilizing Smo CRD inhibitors, exploiting the “rheostat” characteristic of OPCs, could offer a safer approach to developing effective promyelinating drugs. This approach provides specificity, potential for overcoming resistance, and adaptability for various therapeutic applications. Further research and development are likely to improve its efficacy and applicability in clinical settings.

## Data Availability

The original contributions presented in the study are included in the article/Supplementary material, further inquiries can be directed to the corresponding authors.
