# Supplementary material for: Pharmacological targeting of smoothened receptor cysteine-rich domain by Budesonide promotes in vitro myelination
Source: Front Mol Neurosci. 2024 Oct 15;17:1473960. doi: 10.3389/fnmol.2024.1473960 (PMC11518828; doi:10.3389/fnmol.2024.1473960)
Supplement: Supplementary file 1 [file Data_Sheet_1.docx]

Supplementary Material

# Supplementary Data


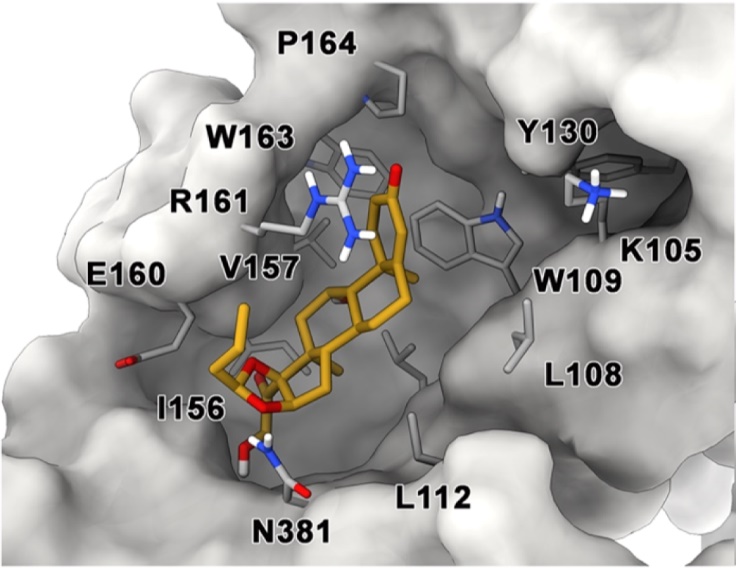


**Supplementary Figure 1**. Docking-predicted binding pose of Budesonide at the hSmo cysteine-rich domain (CRD; PDB: 5L7). The protein is depicted as silver surface. Budesonide and the interacting residues are highlighted in orange, magenta and grey sticks, respectively.


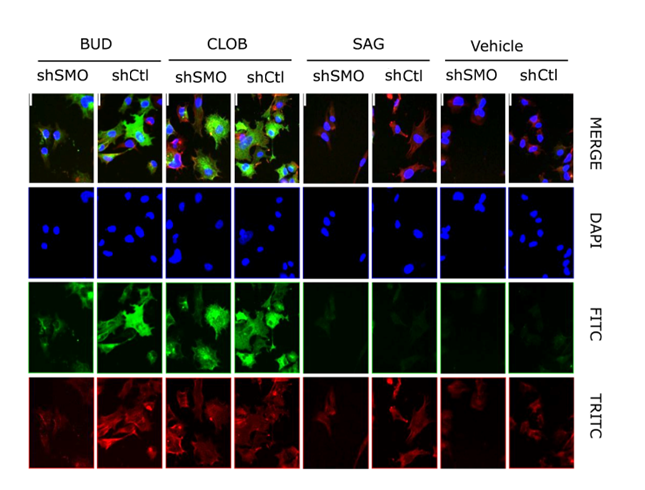


**Supplementary Figure 2.** MBP levels are reduced in Oli-neuM cells silences for Smo (shSMO) compared to control cell line (shCtl). Representative images of IF analysis of Oli-neuMshSMO (shSMO) and Oli-neuMCtl cell lines (shCtl) under treatment with 10 µM Budesonide (BUD), 10 µM Clobetasol (CLOB), 5 µM SAG (SAG), or DMSO < 0.5% (Vehicle) in DM medium for 48 h. After fixation anti-MBP Ab (FITC), Phalloidin (TRITC), Hoechst (DAPI) were used to detect MBP, F-Actin and Nuclei, respectively. ScanR software (3.0 Olympus) was used for image visualization and data analyses shown in the text (Del Giovane et al., 2022). Scale bar =10 µm


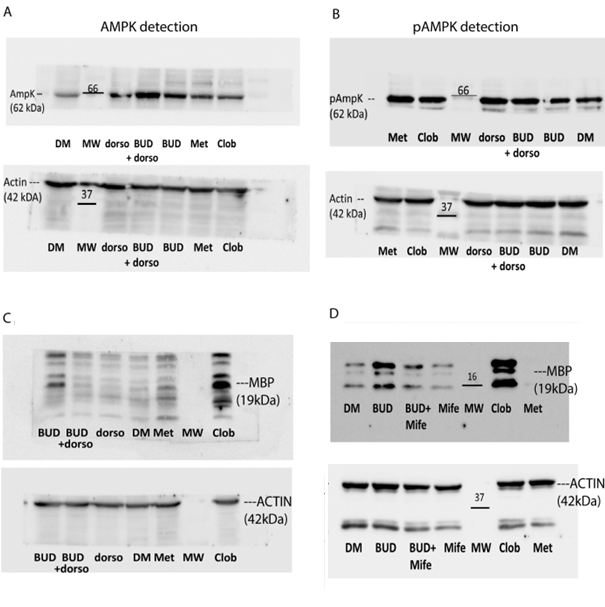
**Supplementary Figure 3. Western blotting Raw data**

Panels A and B: Western Blotting Row data images shown in Figure 5A;

Panel C: Western Blotting Row data images shown in Figure 5B;

Panel D: Western Blotting Row data images shown in Figure 6A.


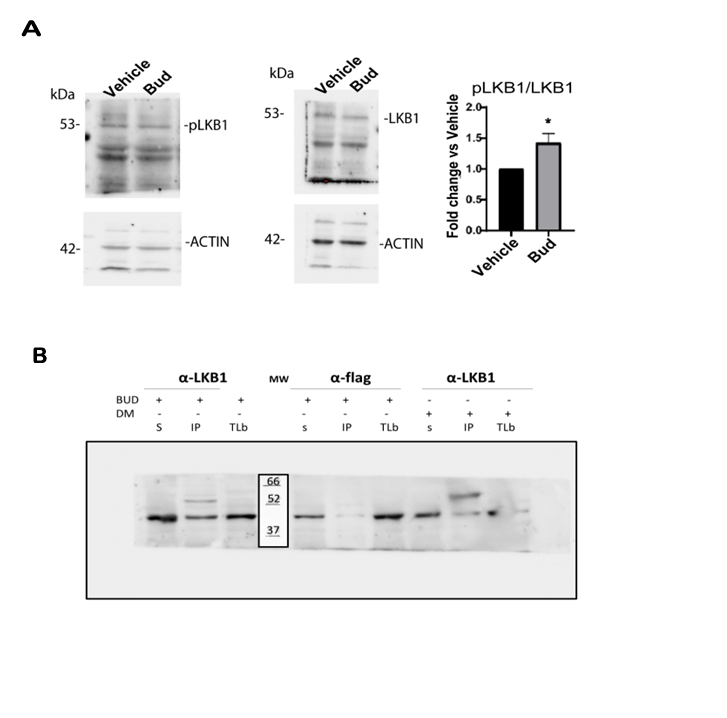


**Supplementary Figure 4. Western Blotting** Raw data LKB Total lysate quantification and Immunoprecipitation.

Panel A) Raw data of IB shown in Figure 5C

Panel B) Raw data of Immunoprecipitation analyses shown in Figure 5D


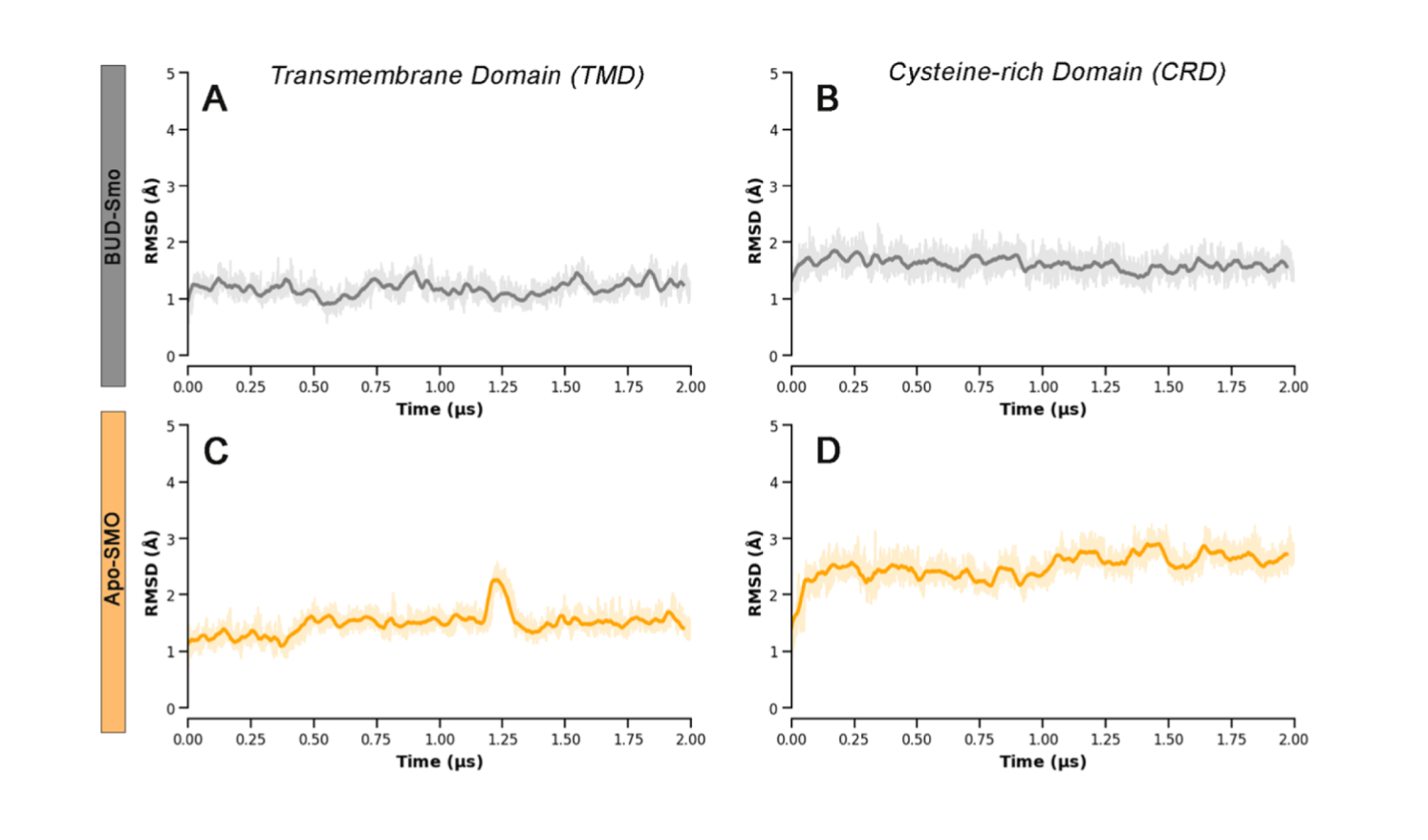


**Supplementary Figure 5.** RMSD of the CRD and TMD computed for the BUD-hSmo (A-B) and apo-Smo (C-D) systems with respect to the starting conformation. Prior to RMSD calculations, the trajectories were aligned on the TMD (A, C) and CRD (B, D) Ca atoms.
